# Very Low Resource Digital Implementation of Bioimpedance Analysis †

**DOI:** 10.3390/s19153381

**Published:** 2019-08-01

**Authors:** Fabien Soulier, Achraf Lamlih, Vincent Kerzérho, Serge Bernard, Tristan Rouyer

**Affiliations:** 1 LIRMM, CNRS, University Montpellier, 34095 Montpellier, France; 2 MARBEC, Ifremer, University Montpellier, 34203 Sète, France

**Keywords:** bioimpedance spectroscopy, multi-frequency, digital processing

## Abstract

Bioimpedance spectroscopy consists of measuring the complex impedance of biological tissues over a large frequency domain. This method is particularly convenient for physiological studies or health monitoring systems. For a wide range of applications, devices need to be portable, wearable or even implantable. Next generation of bioimpedance sensing systems thus require to be implemented with power and resource savings in mind. Impedance measurement methods are divided into two main categories. Some are based on “single-tone” signals while the others use “multi-tone” signals. The firsts benefit from a very simple analysis that may consist of synchronous demodulation. However, due to necessary frequency sweep, the total measurement may take a long time. On the other hand, generating a multi-frequency signal allows the seconds to cover the whole frequency range simultaneously. This is at the cost of a more complex analysis algorithm. This makes both approaches hardly suitable for embedded applications. In this paper, we propose an intermediate approach that combines the speed of multi-tone systems with a low-resource analysis algorithm. This results in a minimal implementation using only adders and synchronous adc. For optimal performances, this small footprint digital processing can be synthesized and embedded on a mixed-mode integrated circuit together with the analog front-end. Moreover, the proposed implementation is easily scalable to fit an arbitrary frequency range. We also show that the resulting impact on noise sensitivity can be mitigated.

## 1. Introduction

Bioimpedance spectroscopy consists of measuring the complex impedance of biological tissues over a large frequency domain [1]. This method is convenient in particular for studying body composition [2], blood characterization [3] and even cancer detection [4]. This wide range of applications makes it suitable as a part of health monitoring systems. Today’s self-monitoring devices tend to be portable, wearable or even implantable. Next, generation bioimpedance sensing systems thus require to be implemented with power and resource savings in mind.

Impedance measurement methods are divided into two main categories. Some are based on “single-tone” signals while the others use “multi-tone” signals. The firsts use a pure frequency sine wave to make the measurement [5,6,7,8]. They benefit from a very simple analysis that can consist of synchronous demodulation or sampling. However, the operation must be repeated for each frequency over the domain of interest. Due to this necessary frequency sweep, the total measurement may take a long time. On the other hand, generating a multi-frequency signal allows the analysis to cover the whole frequency range simultaneously [9,10,11]. This is at the cost of a more complex analysis algorithm (discrete cosine transform—dct, typically). Unfortunately, both methods result in excess power consumption: a long time of measurement for single-tone frequency sweep, hardware and computational resources for multi-tone. This makes both approaches hardly suitable for embedded applications. In 2008, Ronk and Toomessoo proposed an implementation of bioimpedance measurement using multi-frequencies on a field-programmable gate array (fpga) [12]. Their method is similar to dct, but use square-waves instead of sines. This avoids the need of multiplication in the processing.

Some intermediate methods of frequency analysis have been developed with resource savings in mind. They combine the speed of multitone measurement with a much simpler analysis algorithm than dct or fast Fourier transform (fft). For instance, Goertzel filters can be used to calculate frequency components [13]. These methods are particularly useful for applications like the built-in self test of integrated circuits [14].

Last year, we presented a similar approach with further simplifications thanks to the power-of-two frequency distribution [15] at the International Conference on Sensing Technology (icst 2018, Limerick, Ireland). The present paper proposes a slightly improved implementation of the concept resulting in a reduced (divided by two) clock frequency in most of the digital circuitry. As a consequence, the circuit itself avoids a few frequency dividers, all resulting in further power savings. Compared to the conference paper, the present article also adds a new frequency domain analysis of the concept, studies the noise impact on impedance values and gives an extended bibliography.

The rest of the paper is organized as follows. First, we consider some a priori facts about bioimpedance. Those allow us to simplify the resolution needs for a bioimpedance-specific sensor compared to a generic purpose impedance analyzer. Then, we compare classical approaches to impedance analysis for sparse logarithmic frequency distribution and we propose a new methodology. In the fourth section, we analyze the proposed method based on synchronous sampling demodulation and present the processing steps. The fifth section gives the proposal of a digital implementation of the algorithm using only adders and frequency dividers. In the sixth section, we study the impact of noise on impedance estimation. Finally, the last section opens some discussions about the limits of the proposed approach.

## 2. Conceptualization

**Figure 1:**
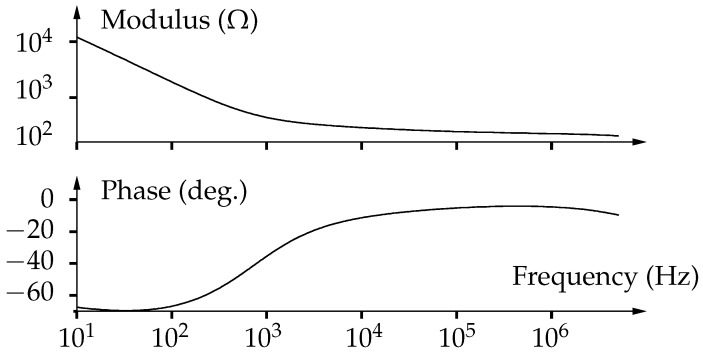
Impedance of bluefin tuna muscle measured using a digital impedance analyzer (*MFIA* from Zurich Instruments AG, Switzerland).

Let’s put into evidence some general characteristics of bioimpedance. All biological tissues present similar frequency behaviors. Typically, the impedance is globally decreasing, with one or several relaxation domains [16] as we can see in Figure 1. Variations are smooth, with noticeably no resonance and only require sparse frequency resolution.

Typical bioimpedance can be modeled with resistors, capacitors and so-called *constant phase elements* (cpe) of impedance

(1) $$\begin{array}{ccc} Z_{\mathrm{CPE}}\left(f\right) & =\frac{1}{q_{0}{\left(2i\pi f\right)}^{\alpha }}, & \mathrm{with}\;q_{0}\;\mathrm{arbitrary}\;\mathrm{parameter},\;\mathrm{and}\;0<\alpha <1. \end{array}$$

**Figure 2:**
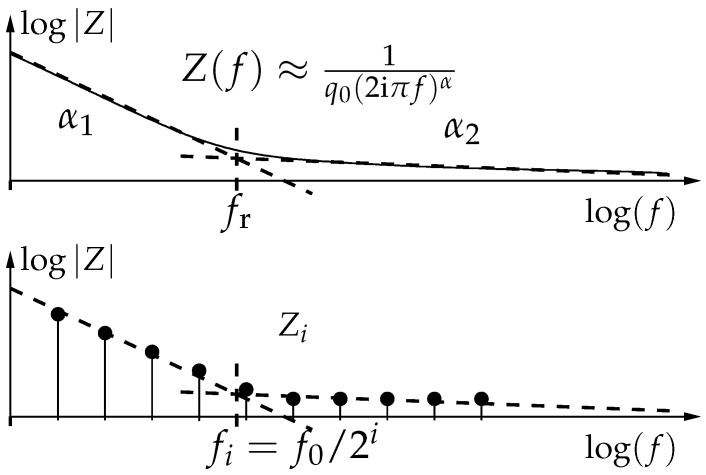
Bioimpedance modeling by constant phase elements (cpe). Two domains and the transition (relaxation) frequency are highlighted (**top**). Logarithmic sampling of the bioimpedance measurements for parameter estimation (**bottom**).

Physiological parameters (fat content, body water, tissue characteristics) are related to objective markers such as: relaxation frequencies, amplitude, and phase of cpe. Estimating such values is better achieved with logarithmically growing frequencies, as illustrated Figure 2. Designing a bioimpedance-specific sensor, we can adopt a frequency distribution of the form

(2) $$\begin{array}{ccc} f_{i} & =\frac{f_{0}}{2^{i}}, & 0\leq i<n_{f}, \end{array}$$

for the multitone excitation signal. This gives $n_{f}$ frequencies with a maximum of $f_{0}$ and a resolution of $\log_{2}\left(10\right)\approx 3.32$ points per decade.

## 3. Methodology

The basic idea behind the proposed method of impedance estimation is the use of coherent sampling together with a multitone excitation signal. Classical approaches either use coherent sampling of a single-tone (pure frequency sine) or Fourier analysis (sine values multiplications) of a multi-tone signal.

**Figure 3:**
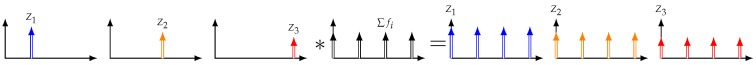
Conceptual representation of impedance spectroscopy based on single-frequency excitation and coherent sampling in frequency domain.

The first case is summarized in Figure 3 where analysis is performed by down-sampling, then low-pass filtering the voltage signal. In the frequency domain, it consists of convoluting the acquired signal by a Dirac comb, then estimating the dc (zero-frequency) content. The signal must be acquired for at least one period of the lowest frequency signal, i.e., $\frac{2^{n_{f}-1}}{f_{0}}$. Because the operation must be repeated for each frequency, the total acquisition time reaches $n_{f}\times \frac{2^{n_{f}-1}}{f_{0}}$.

**Figure 4:**
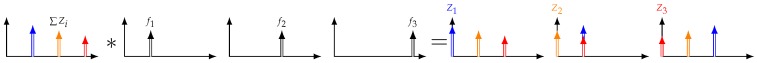
Conceptual representation of impedance spectroscopy based on multi-frequency excitation and Fourier analysis in frequency domain.

The second case is summarized in Figure 4 where analysis is performed by multiplying the multi-frequency voltage signal by pure sine values, then low-pass filtering. In the frequency domain, it consists of convoluting the acquired signal by a unique Dirac, then estimating the dc content. In this case, the signal still must be acquired for at least the inverse of the lowest frequency, i.e., $\frac{2^{n_{f}-1}}{f_{0}}$), but only one time.

Coherent sampling only requires one addition per period, and no multiplication. Thus, the total number of additions is $\sum_{i=0}^{n_{f}-1}2^{i}=2^{n_{f}}-1$. This number must be multiplied by two to get real and imaginary parts of the impedance.

As said before, bioimpedance spectroscopy can be performed using a somehow sparse number of frequency points. Therefore, dct reveals itself to be more appropriate than the fft algorithm. The processing requires as additions and multiplications as the number of samples, $2^{n_{f}}$ (with the adc at the Shannon rate, $2f_{0}$), repeated for each of the $n_{f}$ frequencies of interest. Again, this number must be multiplied by two for real and imaginary parts, i.e., $n_{f}\times 2^{n_{f}+1}$.

**Figure 5:**
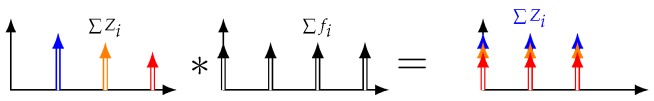
Conceptual representation of impedance spectroscopy based on multi-frequency excitation and coherent sampling in frequency domain.

Mixing coherent sampling with multi-tone excitation results in short acquisition time while avoiding the need for multipliers (see Table 1). This method should be implemented using the lowest power consumption. Unfortunately, Figure 5 shows that aliasing occurs in this case at null frequency, making it harder to process. However, we show in the following section that it is still possible to discriminate valuable information from the measured dc component.

## 4. Formal Analysis

**Figure 6:**
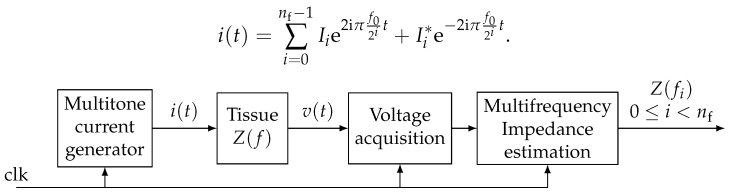
Principle of multi-tone synchronous bioimpedance sensing system.

The chosen principle of bioimpedance sensing is illustrated in Figure 6. With the above considerations, generated current i(t) can be expressed as the superposition of $n_{f}$ sine waves. Current being real, hermitian symmetry applies to complex amplitudes ($I_{-i}=I_{i}^{*}$) such that

(3) $$i\left(t\right)=\sum_{i=0}^{n_{f}-1}I_{i}e^{2i\pi \frac{f_{0}}{2^{i}}t}+I_{i}^{*}e^{-2i\pi \frac{f_{0}}{2^{i}}t}.$$

For the sake of simplicity, we can let all $I_{i}$ be equal to $\frac{1}{2}$ (normalized amplitudes and null phase for all frequency components). If not so, we can get back to this case by resistor calibration:

(4) $$i\left(t\right)=\frac{1}{2}\sum_{i=0}^{n_{f}-1}e^{2i\pi \frac{f_{0}}{2^{i}}t}+e^{-2i\pi \frac{f_{0}}{2^{i}}t}.$$

Let us express the Fourier transform I(f)=F{i(t)} in the frequency domain

(5) $$I\left(f\right)=\frac{1}{2}\sum_{i=0}^{n_{f}-1}\delta \left(f-\frac{f_{0}}{2^{i}}\right)+\delta \left(f+\frac{f_{0}}{2^{i}}\right),$$

with δ(t) denoting the Dirac distribution. We can deduce from the last equation the voltage expression V(f)=F{v(t)} in the sinusoidal steady state:

(6) $$\begin{array}{cc} V(f) & =Z\left(f\right)I\left(f\right)=\frac{1}{2}\sum_{i=0}^{n_{f}-1}Z_{i}\delta \left(f-\frac{f_{0}}{2^{i}}\right)+Z_{i}^{*}\delta \left(f+\frac{f_{0}}{2^{i}}\right), \end{array}$$

with $Z_{i}=Z\left(f_{i}\right)$ and $Z_{i}^{*}=Z\left(f_{-i}\right)$.

The objective of the following processing is to extract the values $Z_{i}=Z\left(f_{i}\right)$ from the acquired v(t) signal. The main idea is to use synchronous sampling demodulation. Indeed, $Z(f_{0})$ can be obtained by sampling v(t) at the higher frequency $f_{0}$ and by low-pass filtering (averaging) the resulting samples. However, sampling the signal at lower frequencies results in aliasing. Let us explicitly express the result of sampling v(t) at $f_{j}=\frac{f_{0}}{2^{j}}$. In the frequency domain, sampling is equivalent to a convolution product by a Dirac comb:

(7) $$V\left(f\right)*\sum_{k\in Z}\delta \left(f-k\frac{f_{0}}{2^{j}}\right)=\frac{1}{2}\sum_{k\in Z}\sum_{i=0}^{n_{f}-1}Z_{i}\delta \left(f-f_{0}\left(\frac{k}{2^{j}}+\frac{1}{2^{i}}\right)\right)+Z_{i}^{*}\delta \left(f-f_{0}\left(\frac{k}{2^{j}}-\frac{1}{2^{i}}\right)\right).$$

We isolate from the last equation the dc component $S_{j}$, i.e., the coefficient of δ(f). For all *i* between 0 and *j*, there are two integers $k=\pm 2^{j-i}$ that satisfy

(8) $$\frac{1}{2^{i}}\pm \frac{k}{2^{j}}=0.$$

Summing all contributions, we get

(9) $$S_{j}=\frac{1}{2}\sum_{i=0}^{j}\left(Z_{i}+Z_{i}^{*}\right)=\sum_{i=0}^{j}ℜ\left(Z_{i}\right).$$

In the same manner, we can get the quadrature components by delaying the sample time by a quarter of period $\frac{2^{j}}{4f_{0}}$:

(10) $$V\left(f\right)*\sum_{k\in Z}\delta \left(f-k\frac{f_{0}}{2^{j}}\right)e^{-2i\pi \frac{2^{j}}{4f_{0}}f}=\frac{1}{2}\sum_{k\in Z}\sum_{i=0}^{n_{f}-1}Z_{i}\delta \left(f-f_{0}\left(\frac{k}{2^{j}}+\frac{1}{2^{i}}\right)\right)e^{-i\pi \frac{k}{2}}+Z_{i}^{*}\delta \left(f-f_{0}\left(\frac{k}{2^{j}}-\frac{1}{2^{i}}\right)\right)e^{-i\pi \frac{k}{2}}.$$

Again, for $k=\pm 2^{j-i}$, we get the dc component

(11) $$Q_{j}=\frac{1}{2}\sum_{i=0}^{j}Z_{i}e^{-i\pi \frac{-2^{j-i}}{2}}+Z_{i}^{*}e^{-i\pi \frac{2^{j-i}}{2}}.$$

If j≥2, we can isolate the terms corresponding to i=j and i=j−1:

(12) $$Q_{j}=\frac{1}{2}\sum_{i=0}^{j-2}Z_{i}e^{i\pi 2^{\left(j-i-1\right)}}+Z_{i}^{*}e^{-i\pi 2^{\left(j-i-1\right)}}+\frac{1}{2}\left(Z_{j-1}e^{i\pi }+Z_{j-1}^{*}e^{-i\pi }\right)+\frac{1}{2}\left(iZ_{j}-iZ_{j}^{*}\right).$$

Thus,

(13) $$Q_{j}=\sum_{i=0}^{j-2}ℜ\left(Z_{i}\right)-ℜ\left(Z_{j-1}\right)-ℑ\left(Z_{j}\right).$$

From Equations (9), (11) and (13), we can recursively calculate real and imaginary parts of $Z_{j}$.

(14) $$\begin{array}{cccc} ℜ(Z_{0}) & =S_{0}, & ℑ(Z_{0}) & =-Q_{0}, \end{array}$$

(15) $$\begin{array}{cccc} ℜ(Z_{1}) & =S_{1}-S_{0}, & ℑ(Z_{1}) & =-Q_{1}-S_{0}, \end{array}$$

(16) $$\begin{array}{cccc} ℜ(Z_{j}) & =S_{j}-S_{j-1}, & ℑ(Z_{j}) & =2S_{j-2}-S_{j-1}-Q_{j}. \end{array}$$

## 5. Numerical Simulation

In order to validate the formal analysis described in Section 4, we have performed some numerical simulations using the open-source computational software Scilab. The code provided in Listing 1 tests the algorithm for eight frequencies and a sample rate of 1MHz. To make results more realistic, we use measurements from tuna muscle bioimpedance (Figure 1) as the reference model. As expected, estimated values by decimation–accumulation and post-processing are in perfect agreement with the reference (Table 2).

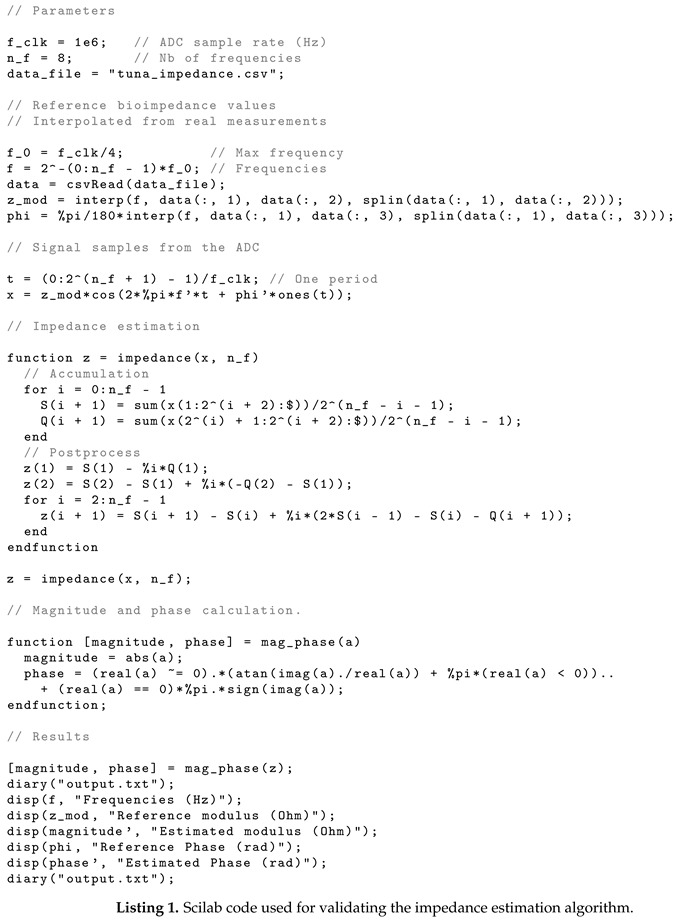



## 6. Implementation

**Figure 7:**
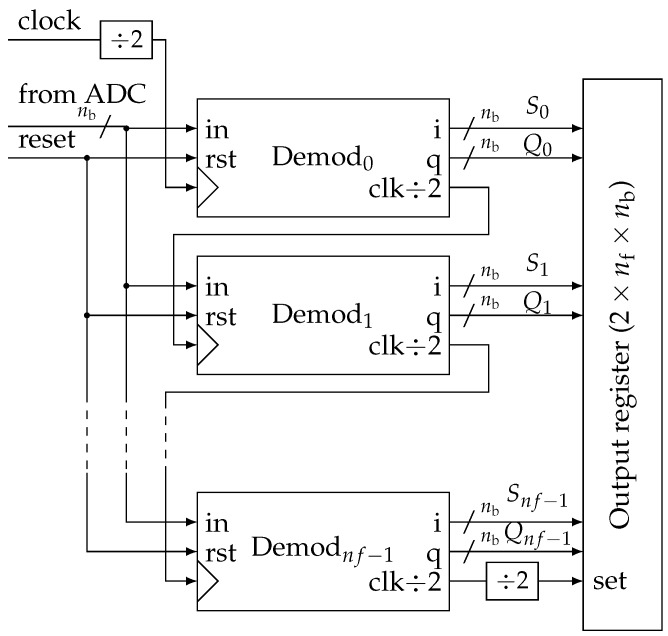
Overview of the parallel implementation of the impedance estimation algorithm.

The main advantage of the proposed method is the simplicity of its digital implementation. Like classical synchronous demodulation, it only requires an adc sharing the same clock as the signal generation (Figure 6). Samples are fed by the adc at the rate of $f_{\mathrm{clk}}=4f_{0}$. Then, they are re-sampled at each frequency $f_{i}$ composing the multitone signal. The extraction of the dc component can be easily done by averaging the samples over a period of the whole multitone signal. Then, sample decimation is performed between each stage by dividing the frequency by two (Figure 7).

**Figure 8:**
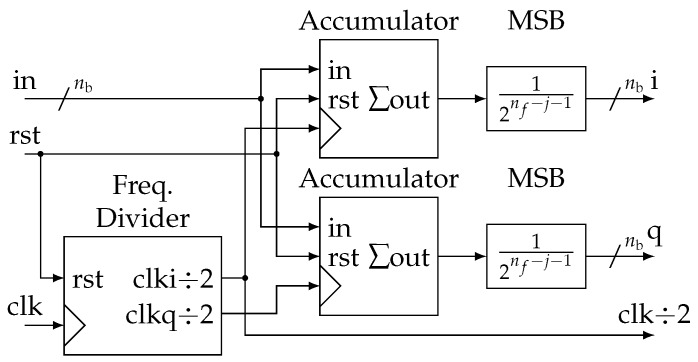
Details of the “Demodulator” generic block.

**Figure 9:**
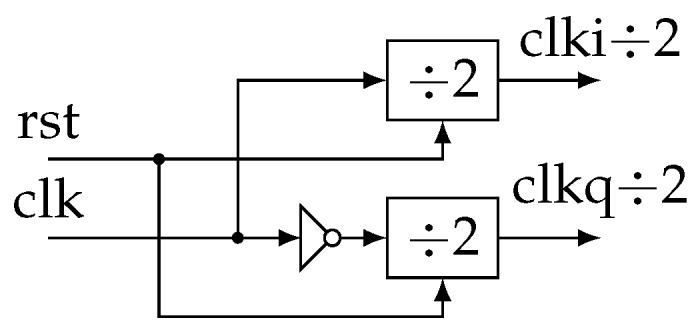
Details of the “Freq. Divider” block.

**Figure 10:**
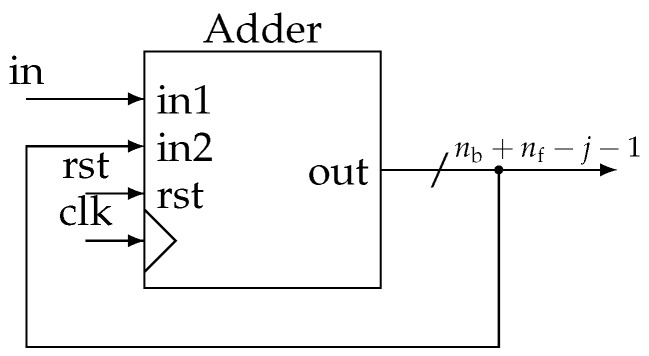
Details of the “Accumulator” block.

Practically, these functions are grouped in a block called *Demod* for demodulator that can be easily instantiated from a generic description (Figure 8). In more detail, at each stage, we generate two clock signals in quadrature (Figure 9). They control two identical accumulators (Figure 10). Samples are accumulated at the rate corresponding to a particular frequency $f_{i}$. The adder size is adapted to avoid overflow. Then, dividing by $\frac{1}{2^{n_{f}-j-1}}$ is done by keeping only the $n_{b}$ most significant bits (msb). Thus, all $S_{j}$ and $Q_{j}$ accumulation are eventually encoded with the same precision.

Values of $S_{j}$ (in-phase) and $Q_{j}$ (in-quadrature) are saved in the output register at the end of the global period ($\frac{2^{n_{f}}}{f_{0}}$). This is achieved using a simple frequency divider on the clock output of the last demodulator. We choose not to embed the computation of actual real and imaginary parts of the impedance as described by Equations (14) to (16). It can be done without transmission penalty (same size of data) as a post-process. The objective is to keep the digital circuit as simple as possible in a low-power constraint.

It must be noticed that the proposed algorithm is mathematically equivalent to a bank of finite impulse response (fir) filters. This is in particular made explicit in the implementation proposed by [17] that uses a similar multiplication-less approach. However, the straightforward implementation of such filters increases a lot the complexity of embedded computations and lacks genericity due to differing coefficients of each filter.

In our design, the parallel architecture can smoothly scale with the number $n_{f}$ of frequency components. Moreover, each stage only uses two adders together with a few flip-flops to divide the frequency. For optimal performances, it is totally realistic to embed this digital processing directly with the analog front-end (current source and sensing amplifier) in a mixed-mode application-specific integrated circuit (asic).

## 7. Noise Sensitivity

The proposed architecture raises the question of the effect of noise on measurements. In particular, low frequencies suffer from averaging low numbers of samples. The worst occurring for $f_{n_{f}-1}=\frac{f_{0}}{2^{n_{f}-1}}$ where only one sample is used to calculate each of $S_{n_{f}-1}$ and $Q_{n_{f}-1}$. In this section, we show that, in the case of bioimpedance spectroscopy, this effect can be mitigated.

We now model perturbations by additive white noise *n*. Let $\sigma^{2}$ be the noise power. Voltage samples from the adc can be written v[i]+n[i]. At the end of the period, in-phase and quadrature accumulators of rank *j* have summed $2^{n_{f}-j-1}$ samples:

(17) $$\begin{array}{cc} \hat{S_{j}} & =\frac{1}{2^{n_{f}-j-1}}\sum_{i=0}^{2^{n_{f}-j-1}-1}\left(v\left[2^{j+2}i\right]+n\left[2^{j+2}i\right]\right), \end{array}$$

(18) $$\begin{array}{cc} \hat{Q_{j}} & =\frac{1}{2^{n_{f}-j-1}}\sum_{i=0}^{2^{n_{f}-j-1}-1}\left(v\left[2^{j}\left(4i+1\right)\right]+n\left[2^{j}\left(4i+1\right)\right]\right). \end{array}$$

From Equation (15), the estimated value of the impedance real part is

(19) $$ℜ\left({\hat{Z}}_{j}\right)={\hat{S}}_{j}-{\hat{S}}_{j-1}.$$

This combination contains $2^{n_{f}-j-1}$ noise samples, thus the signal power

(20) $$P\left\{ℜ\left({\hat{Z}}_{j}\right)\right\}=ℜ{\left(Z_{j}\right)}^{2}+2^{n_{f}-j-1}\frac{\sigma^{2}}{{\left(2^{n_{f}-j-1}\right)}^{2}}=ℜ{\left(Z_{j}\right)}^{2}+\frac{\sigma^{2}}{\left(2^{n_{f}-j-1}\right)}.$$

In the same manner,

(21) $$ℑ\left({\hat{Z}}_{j}\right)=2{\hat{S}}_{j-2}-{\hat{S}}_{j-1}-{\hat{Q}}_{j}.$$

Consider $2^{n_{f}-j+1}$ noise samples, thus the power

(22) $$\begin{array}{c} P\left\{ℑ\left({\hat{Z}}_{j}\right)\right\}=ℑ{\left(Z_{j}\right)}^{2}+2^{n_{f}-j+1}\frac{\sigma^{2}}{{\left(2^{n_{f}-j+1}\right)}^{2}}=ℑ{\left(Z_{j}\right)}^{2}+\frac{\sigma^{2}}{\left(2^{n_{f}-j+1}\right)}. \end{array}$$

Now, the signal-to-noise ratio (snr) can be expressed as

(23) $$\mathrm{SNR}=2^{n_{f}-j-1}\frac{ℜ{\left(Z_{j}\right)}^{2}}{\sigma^{2}}.$$

Because we study bioimpedance, Z(f) can be assimilated to cpe. Using Equation (1), we assume that α≈1/2:

(24) $$ℜ{\left(Z_{j}\right)}^{2}=\frac{1}{2}{\left|Z\left(\frac{f_{0}}{2^{j}}\right)\right|}^{2}=\frac{2^{j}}{2q_{0}\pi f_{0}}.$$

Leading to the signal-to-noise ratio

(25) $$\mathrm{SNR}=\frac{2^{n_{f}-2}}{\sigma^{2}q_{0}\pi f_{0}}.$$

**Figure 11:**
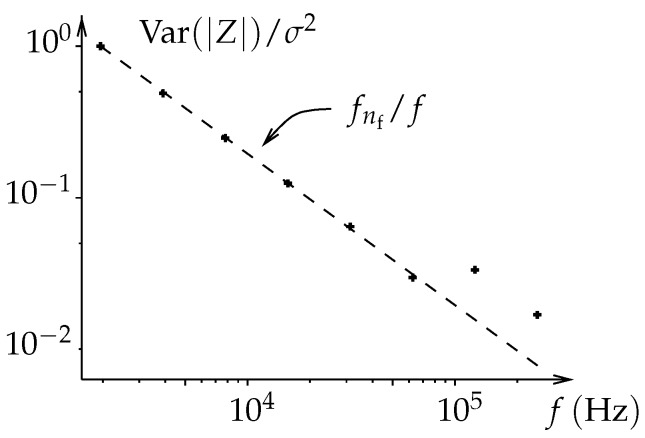
Variance of the impedance measurements exhibiting 1/f evolution. The variance is numerically estimated over 1000 random trials where gaussian noise has been added to adc datas. Variance is normalized by noise power ($\sigma^{2}$).

We can notice that the expression does not depend on rank *j*. Obviously, the same conclusion applies for the imaginary part. Therefore, with the previous assumptions, the snr is almost constant along the whole frequency range. This is due to typical bioimpendances decreasing at the same rate as the squared root variance of the proposed estimator (Figure 1). A numerical simulation has been performed to illustrate this behavior (Figure 11).

Finally, in the case where the impedance does not decrease as $1/\sqrt{f}$, a solution may be to adapt the generated current signal by increasing the amplitudes for frequencies where the snr worsens.

## 8. Discussion

In the presented bioimpedance spectroscopy system, all frequency components are processed in parallel. Thus, mismatch between channels might impact output accuracy. However, with the proposed architecture, all signal paths (real, imaginary, for each frequency) are fed from the same ADC. Considering a particular channel, data are just sub-samples of a unique sample flow. All further processing being digital, the only source of mismatch appears to be the ADC clock jitter. Under the reasonable assumption that jitter is statistically independent from the sub-sampling operation, we hope that this effect will be moderated by the accumulation operation. From this point of view, jitter may be treated as an additional noise (or error) on sample values.

Another concern may be the very-low frequency noise due to amplifier offset, electronics 1/f noise, electrode interface potential, etc. It would induce dc component that would impact all $S_{j}$ and $Q_{j}$ values. However, it appears relatively easy to get rid of this effect by averaging all samples at $f_{\mathrm{clk}}$, i.e., before decimation and subtracting this dc value during post-processing.

The last issue could be the fixed resolution due to frequency distribution as powers of $\frac{1}{2}$. If more resolution is needed, a non-power-of-two frequency division can be introduced, e.g., $\frac{f_{\mathrm{clk}}}{3}$ and then used instead of $f_{\mathrm{clk}}$ in a duplicated structure. Of course, expressions of $S_{j}$ and $Q_{j}$ will be affected and need to be re-evaluated. However, only post-processing computation will be modified.

## 9. Conclusions

We have proposed a very efficient implementation of multitone analysis for bioimpedance sensing. Thanks to general characteristics of bioimpedance, we have chosen a logarithmic frequency distribution. With this hypothesis, we can explicit the aliasing resulting from successive synchronous demodulations and decimations. This expression can be used to post-process the measurements and estimate real and imaginary parts of the impedance for each frequency. The digital implementation consists only in accumulators and frequency dividers and does not make any use of multipliers. Moreover, the structure can be easily scaled to wider frequency ranges. Despite expected degradation of snr for low frequencies, we show that this effect is mitigated by the increase of impedance in this range.

## Figures and Tables

**Table 1:** Expected characteristics of impedance estimation methods.

			(Proposed Method)
	Single Freq.	Multi Freq.	Multi Freq.
	& Coherent Sampling	& DCT	& Coherent Sampling
Acquisition time (mini.)	$n_{f}\times \frac{2^{n_{f}-1}}{f_{0}}$	$\frac{2^{n_{f}-1}}{f_{0}}$	$\frac{2^{n_{f}-1}}{f_{0}}$
Additions	$2\times (2^{n_{f}}-1)$	$n_{f}\times 2^{n_{f}+1}$	$2\times (2^{n_{f}}-1)$
Multiplications	0	$n_{f}\times 2^{n_{f}+1}$	0

**Table 2:** Results of the numerical simulation performed with Scilab compared with reference values.

Frequencies	Reference	Estimated	Reference	Estimated
(Hz)	Modulus (Ohm)	Modulus (Ohm)	Phase (Rad)	Phase (Rad)
250,000	235.55872	235.55872	−0.0740422	−0.0740422
125,000	242.33682	242.33682	−0.0852266	−0.0852266
62,500	251.06561	251.06561	−0.1040394	−0.1040394
31,250	262.43457	262.43457	−0.1302557	−0.1302557
15,625	277.09086	277.09086	−0.1660522	−0.1660522
7812.5	295.96917	295.96917	−0.2183331	−0.2183331
3906.25	321.75482	321.75482	−0.3006170	−0.3006170
1953.125	362.5306	362.5306	−0.4326722	−0.4326722

## Abbreviations

The following abbreviations are used in this manuscript:

ADC	Analog to Digital Converter
ASIC	Application-Specific Integrated Circuit
CPE	Constant Phase Element
DCT	Discrete Cosine Transform
FIR	Finite Impulse Response filter
FFT	Fast Fourier Transform
FPGA	Field-Programmable Gate Array
MSB	Most Significant Bit
SNR	Signal-to-Noise Ratio
